# Values Evolution in Human Machine Relations: Grounding Computationalism and Neural Dynamics in a Physical a Priorism of Nature

**DOI:** 10.3389/fnhum.2021.649544

**Published:** 2021-05-11

**Authors:** Denis Larrivee

**Affiliations:** ^1^Mind and Brain Institute, School of Medicine, University of Navarra, Pamplona, Spain; ^2^Department of Arts and Sciences, Loyola University, Chicago, IL, United States

**Keywords:** computationalism, ethical parity, posthumanism, extended mind theory, brain computer interface, motor image, action attribution, cyborg

## Introduction

“*There is a demand for more and more sophisticated social robots. The ideal of many engineers is to produce machines indistinguishable from humans, on the level of behavior or appearance….” (Campa, 2016)*.

Artificial intelligence and its companion technology robotics promise to revolutionize human machine relations through their capabilities for analyzing, interpreting, and executing human action (Institute of Electrical and Electronic Engineers, 2017). While stimulating excitement as well as concern (Bostrom, 2014), these capabilities have also invited reflection on the ethics and values guiding technology development (Calo, 2016). Factors that induce value evolution are of interest, therefore, for influencing the forms the technology may adopt.

In broad terms these are seen to operate at two levels: (1) by epistemological inference, often through neuroscientific observation—humans are like machines (McCulloch and Pitts, 1943; Fodor, 1975; Marr and Poggio, 1976; Marr, 1982; Piccinini, 2004; Yuste, 2010) and (2) by ontological predication, that is, as an imputed analog of human meta properties—machines are like humans (Hornyak, 2006; Kitano, 2006; Sabanovic, 2014). Due to their design intent of reducing the onus of human intervention, AI devices are increasingly given over to servicing a spectrum of human needs, from lower order motoric assistance to higher order computational and social functions, e.g., living assistance companions and work colleagues (Sabanovic, 2014); accordingly, they invite analogy at multiple levels.

Simulation of higher order cognition, especially, is regarded as driving value attribution—here understood as an intrinsic ground for rights and ethical entitlement (Rothaar, 2010)—which flows from ontological inferences about the technology's operational semblance to human cognition. That is, through replication of these uniquely human abilities, there is a growing ontological incursion in the technology, which propels value evolution under the guise of simulating ontological equivalence. Breazeale's Kismet robot, for instance, explores not merely the social gestures essential to promoting human machine interactions but also the construction of human social intelligence and even what it means to be human (Breazeal, 2002; Calo, 2016).

Simulation thus challenges the traditional value hierarchy placing human beings at the apex of organismal life and grounding ethical, bioethical, and neuroethical praxis, a prioritization that has promoted human flourishing while also restricting harmful intervention into the human being. Rather than emphasizing the centrality of human value, simulation promotes a value architecture that is more inclusive, democratic, and horizontal in scope, a trend recently taken up in ethical parity models (Clark and Chalmers, 1998; Levy, 2011; Chandler, 2013). Seen through the lens of ethical parity, however, simulation poses a multidimensional challenge to an ethical system where value is contingent to the human being, a challenge mediated at the level of the ethical subject, i.e., in the siting of value contingency (Clark and Chalmers, 1998; Levy, 2011), in its theory of ethics (Latour, 1993; Connolly, 2011), i.e., in how ethics is normatively anchored (Latour, 2007), and in ethical praxis (Sgreccia, 2012). In consequence, it modifies ethical mediation as an intentionalized moral enactment, which is framed by a referential ontology.

The pursuit of ethical parity between robotic technology and the human being has highlighted the symbiotic nature of human machine relations (Haraway, 2003; Rae, 2014a). Rather than the merely instrumentalist association identified in Aristotelian and scholastic philosophy, the appropriation of ontological parity motivates a physical reciprocity that lies at the intersection of the human and the machine; that is, behind the human lies hidden the machine, and behind the machine lies the human. Hence, symbiosis is understood to actuate an a priorism that is physically operative at the locus of intersection between the two (Waters, 2006; Onishi, 2011).

Elucidating the philosophical roots of this a priorism is, nonetheless, infrequently considered (Rae, 2014b). While the detection of a physical ‘a priorism’ can be expected to constitute a *meta* valorization of the process of ontological appropriation distinguishing simulation, epistemological sources that may reveal consilience have yet to trace the physical reciprocity invoked by symbiosis to a *meta-*physical ground (Haraway, 2003; Rae, 2014a).

Modern physics, for example, broadly views the world as consisting of individual entities embedded in space time (Esfeld, 2004), a conception rarely considered in human machine, philosophy of science guises and apparently contravened by the sort of symbiosis proposed in their chimeras.

This paper will opine that standard simulation accounts like computationalism trace their understanding of ontology to Heidegger's metaphysical deconstruction of subject/object dichotomies which identified a constitutive a priorism of attribute sharing. Recent integrationist accounts of cognition, however, increasingly evidence a unity structured through the body's engagement in action (Fourneret et al., 2002; Kato et al., 2015; Noel et al., 2018; Wolpaw, 2018); that is, neural architectures reveal an a priorism grounded in the unity of their operation, a finding of relevance for ontology, where actionable behaviors qualify an emergent self.

## Simulation Through Functionalism to Heidegger

“And, in spite of the victory of the new quantum theory, and the conversion of so many physicists to indeterminism de La Mettrie's doctrine that man is a machine has perhaps more defenders than before among physicists, biologists and philosophers; especially in the form of the thesis that man is a computer.” (Popper, 1978).

As Karl Popper notes (Popper, 1978), the thesis that human cognition simulates the computational abilities of machines has propelled the widely held notion that humans share an ontological equivalence with computational machines. Indeed, over the last half century, computationalism—whether classicist, connectionist, or neurocomputating variants—has dominated thinking on cognition. Beginning with McCulloch and Pitts (1943), Karl Lashley (Piccinini, 2004), and others (Fodor, 1975; Marr and Poggio, 1976) this thesis has evolved through several incarnations, notably Marr and Poggio's extension to information processing and Fodor's symbolic, Turing style computation that enabled decision making, perception, and linguistic processing (Fodor, 1975). This latter, particularly, has served to bridge the divide between computational events and functions carried out by the mind; that is, mental functions are understood to be built on computational processes, which link human and machine at the level of capacities operative in the human mind (Putnam, 1999).

Extrapolating from Fodor's transposition, simulation is thereby invoked as a methodological paradigm for arriving at ontological parity. In fact, computationalism implicitly claims the absence of ontological distinction, due to the semblance between commonly shared attributes, an absence that has grounded the physical reciprocity between human and machine highlighted in conceptions of human machine symbioses. Further, the thesis that mental states configure mental functions (Putnam, 1999) views the mind as constitutively functional. Understood this way the mind lacks a unique physical substrate, a conception incompatible with a distinctive ontology. Clark and Chalmer's extended mind hypothesis (Clark and Chalmers, 1998; Levy, 2011), for example, is notably distinguished for the mind's lack of a unique physical origin.

The lack of distinction, however, challenges traditional subject/object dichotomies that view the human in opposition to the machine (Rae, 2010, 2014a), motivating a conception of ontology grounded not in meta features of the world but in each entity's relationship with the other. The imagery of the cyborg, for example, has been evoked precisely for conceptualizing beyond binary oppositions (Haraway, 2003), which would otherwise foreclose the physical reciprocity implied by semblance. As Onishi points out, this emphasis on a least common denominator—a main tenet of the transhumanist vision, for example, is the belief ‘that the worlds’ only underlying and universal feature is information—has the serious ontological consequence of allowing technology, especially neurotechnology in the form of brain, machine permutations, to reshape material existence, including the human body, at will (Chandler, 2013; Rae, 2014a). Such a conception denies the existence of an a prioristic, *meta*-physical structure that grounds physical reality.

For computationalism the machine-human metaphor has gained traction from Heidegger's critique of metaphysical humanism that likewise challenged subject/object dichotomies, but did so at the level of being, a critique subsequently laying the foundation for the “anti”-humanism of structuralist, post-structuralist, and deconstructionist thought (Rockmore, 1995). Heidegger's challenge to the Cartesian metaphysical legacy of binary oppositions (which itself challenged scholastic notions of a priori form and purpose) rooted itself in an understanding of being as that which *enabled* ‘things to be’ rather than that *exhibited* by their reality. His apriorism of a ‘murky’ being (Rae, 2014a), led him to posit the ‘nullity’ that now defines the postmodern subject (Onishi, 2011); hence, in the absence of property predication, the subject must be recreated through his network of external relations. Indeed, much of the fluid, networked understanding incorporated in such thinking emerges from the separation of being from its anchorage in entities, and the ensuing requirement to restructure the entity through network interactions (Latour, 2007; Chandler, 2013).

## Human Action and Dynamic Entities in a Metaphysics of Nature

While Heidegger's critique is crucial for structuring ontological parity between humans and machines by means of a novel metaphysical paradigm of being, this latter is not generally invoked a priori as a meta conception of the physical world. Esfeld (2004), for example, points out that according to the modern mainstream of *meta*-physical thought, the physical world consists of independent and individuated things that are embedded in space–time. These things are individual because they are spatio-temporally unique and entities because they are (a) each the subject of the predication of properties that are (b) qualitatively distinguished from those of all other individuals.

This broad—indeed historical—recognition that the world's physical meta-structure is composed of entities underscores a consensus that individuation characterizes the physical world. By contrast, Heidegger's premise that entities can ‘be’ apart from their qualities leaves open the question of whether being is one or many, thereby denying that individuation is a constitutive feature of reality. Hence, the understanding of individuation has repercussions for how ontology is conceived.

Individuation reveals, especially, that unity is constitutive, not ‘merely’ for property predication, but in so far as what things ‘are’ and the basis for their persistence; hence, in contemporary physical understanding entities are individuated because they are unified. Meta understandings of the physical world, critically, prominently feature an a priori operational dynamic that is a principle of unification, recapitulating Aquinas's dynamic notion of unity in operation (Phelan, 1967; Clark, 2003)

“*Every thing exists for the sake of its operation.”*

Evidenced as a normative standard, what is ‘good’ or ‘bad’ may be evaluated according to its contribution toward the persistence of a ‘thing's’ operation, that is, in its adherence to this principle.

In line with this, living systems notably exhibit a unity autonomously mediated during action execution (Fourneret et al., 2002; Farrer et al., 2004; Jeannerod, 2009); that is, a self-organizing principle functioning as a dynamic locus of action origin. Along an arc of expanding neural complexity and range of action this operational dynamic unifies behavioral performance. At its apex, humans display an ontological crescendo where autonomous actions predicate from a globally distinct, operative order (Rizzolatti and Fabbri-Destro, 2008; Orban and Caruana, 2014); that is, a physical a priorism of unity deployed through operational breadth. Human ontology, thereby, is an emergent qualification defined by unity, operation, and self-presence; hence, an ontology that is subjectively distinct and grounded in the world's a priori features.

This physical a priorism is widely evident, seen, for example, in a panorama of processes generating the unified subject of action:

*A global activity state regulating motor signal delivery (Kato et al.*, *2015**)*

*Mechanisms of total bodily integration in action planning and execution* (Mimica et al., 2018)

*The somatic and multisensory integration of the individual* as the subject of experience (Noel et al., 2018)

*The designation of the body as an agent* of discrete motions (Rizzolatti and Fabbri-Destro, 2008; Jeannerod, 2009; Orban and Caruana, 2014; Brinkers, 2015).

## Humans and Machines in a Physical World

While the semblance of AI and robotics technologies to human cognition motivates a claim to value parity, the failure of its metaphysical roots to account for an evident ontological multiplicity weakens its ethical parity claim. The presence of operationally dynamic entities throughout the physical world evidences instead a principle of individuation recapitulated within an ontological ascent, radically grounding ethical relations between humans and machines in a prioristic features of the natural world.

## Author's Note

Development of sophisticated AI and robotics technologies has motivated claims for their ontological and value parity with humans, due to their purported simulation of human cognitive abilities. Computationalism, for example, is a widely invoked thesis used to explain human cognition and the unique abilities of humans to reason and make decisions. The claim of ontological parity currently underpins advocacy for various forms of human machine chimeras, which identify a physical a priorism in commonly shared attributes, like information, said to ground meta features of physical reality. Tracing this ‘least common denominator’ understanding—frequently invoked in post-humanist thought—to Heidegger's conception of being, however, denies the constitutive presence of a principle of individuation in physical reality. Most modern, as well as historical, conceptions of the physical world, by contrast, recognize the existence of holisms which are distinguished by their properties. Living systems conform to this meta understanding, although distinguished from other entity classes on the basis of a dynamic, operational unity. Neuroscientific observations confirm this dynamic, global unity, which also underpins the computational properties of cognition. This global integration offers a basis for ascribing ontological distinction to humans and for informing ethical values guiding human machine relations.

## Author Contributions

The author confirms being the sole contributor of this work and has approved it for publication.

## Conflict of Interest

The author declares that the research was conducted in the absence of any commercial or financial relationships that could be construed as a potential conflict of interest.

## References

[B1] Bostrom N. (2014). Superintelligence: Paths, Dangers, Strategies. Oxford: Oxford University Press.

[B2] Breazeal C. L. (2002). Designing Sociable Robots. Cambridge, MA: The MIT Press. 10.1007/0-306-47373-9_18

[B3] Brinkers M. (2015). Beyond sensorimotor segregation: on mirror neurons and social affordance space tracking. Cog. Syst. Res. 34, 18–34. 10.1016/j.cogsys.2015.07.002

[B4] Calo R. (2016). Robots as legal metaphors. Harvard J. Law Tech. 30, 209–237.

[B5] Campa R. (2016). The rise of social robots: a review of the recent literature. J. Evol. Tech. 26, 106–113.

[B6] Chandler D. (2013). The world of attachment? the post-humanist challenge to freedom and necessity. Millennium J. Internat Stud. 41, 516–534. 10.1177/0305829813481840

[B7] Clark A. (2003). Natural-Born Cyborgs: Minds, Technologies, and the Future of Human Intelligence. Oxford: Oxford University Press.

[B8] Clark A. Chalmers D. (1998). The extended mind. Analysis 58, 7–19. 10.1093/analys/58.1.7

[B9] Connolly W. (2011). A World of Becoming. London: Duke University Press.

[B10] Esfeld M. (2004). Quantum entanglement and a metaphysics of relations. Studies Hist. Phil. Mod Phys. 35, 601–617. 10.1016/j.shpsb.2004.04.008

[B11] Farrer C. Franck N. Frith C. D. Decety J. Georgieff N. D'Amato T.. (2004). Neural correlates of action attribution in schizophrenia. Psych. Res. 131, 31–44. 10.1016/j.pscychresns.2004.02.004 15246453

[B12] Fodor J. A. (1975). The Language of Thought. Cambridge, MA: Harvard University Press.

[B13] Fourneret P. Vignemont F. A. Franck N. Slachevsky A. Dubois B. Jeannerod M. (2002). Perception of self-generated action in schizophrenia. Cog. Neuropsych. 7, 139–156. 10.1080/13546800143000212 16571532

[B14] Haraway D. (2003). Cyborgs to companion species: reconfiguring kinship in technoscience, in Chasing Technoscience: Matrix for Materiality, eds. Ihde D. Selinger E., (Indianapolis: Indiana University Press), 58–82.

[B15] Hornyak T. N. (2006). Loving the Machine: The Art and Science of Japanese Robots. Tokyo: Kodansha International.

[B16] Institute of Electrical and Electronic Engineers (2017). Ethically Aligned Design. Available online at: https://engagestandards.ieee.org/rs/ (accessed March 26, 2018).

[B17] Jeannerod M. (2009). The sense of agency and its disturbances in schizophrenia: a reappraisal. Exp. Brain Res. 192, 527–532. 10.1007/s00221-008-1533-3 18709365

[B18] Kato S. Kaplan H. S. Schro T. Skora S. Lindsay T. H. Yemini E.. (2015). Global brain dynamics embed the motor command sequence of *Caenorhabditis elegans*. Cell 163, 656– 10.1016/j.cell.2015.09.034 26478179

[B19] Kitano N. (2006). Roboethics – a comparative analysis of social acceptance of robots between the West and Japan. Paper Presented at the Euron Roboethics Atlier, Genoa.

[B20] Latour B. (1993). We Have Never Been Modern. Cambridge, MA: Harvard University Press.

[B21] Latour B. (2007). Reassembling the Social: An Introduction to Actor-Network-Theory. Oxford: Oxford University Press.

[B22] Levy N. (2011). Neuroethics and the extended mind, in The Oxford Handbook of Neuroethics, eds Illes J. Sahakian B.. (Oxford: Oxford University Press). 10.1093/oxfordhb/9780199570706.013.0071

[B23] Marr D. (1982). Vision. Cambridge, MA: MIT Press.

[B24] Marr D. Poggio T. (1976). Cooperative computation of stereo disparity. Science 194, 283–287. 10.1126/science.968482 968482

[B25] McCulloch W. S. Pitts W. H. (1943). A logical calculus of the ideas immanent in nervous activity. Bull. Math. Biophys. 7, 115–133. 10.1007/BF02478259 2185863

[B26] Mimica B. Dunn D. A. Tombaz T. Bojja C. Whitlock J. R. (2018). Efficient cortical coding of 3D posture in freely behaving rats. Science 362, 584–589. 10.1126/science.aau2013 30385578

[B27] Noel J. P. Blanke O. Serino A. (2018). From multisensory integration in peripersonal space to bodily self-consciousness: from statistical regularities to statistical inference. Annals N. Y. Acad. Sci. Special Issue 1426, 146–165. 10.1111/nyas.13867 29876922

[B28] Onishi B. (2011). Tracing visions of the posthuman. Sophia 50, 101–111. 10.1007/s11841-010-0214-4

[B29] Orban G. A. Caruana F. (2014). The neural basis of human tool use. Front. Psychol. 5:310. 10.3389/fpsyg.2014.00310 24782809 PMC3988392

[B30] Phelan G. (1967). The Existentialism of St Thomas, Selected Papers. Toronto: Pontifical Institute of Medieval Studies.

[B31] Piccinini G. (2004). Functionalism, computationalism, and mental contents. Can. J. Phil. 34, 375–410. 10.1080/00455091.2004.10716572

[B32] Popper K. (1978). Of clouds and clocks, in Objective Knowledge: An Evolutionary Approach. 224.

[B33] Putnam H. (1999). The nature of mental states, in *Mind and Cognition: an Anthology, 2nd Edn*. ed. Lycan W. (Malden: Blackwell), 27–34.

[B34] Rae G. (2010). Re-thinking the human: Heidegger, fundamental ontology, and humanism. Human Stud. 33, 23–39. 10.1007/s10746-010-9136-y

[B35] Rae G. (2014a). Heidegger's influence on posthumanism: the destruction of metaphysics, technology, and the overcoming of anthropocentrism. Hist. Human Sci. 27, 51–69. 10.1177/0952695113500973

[B36] Rae G. (2014b). The philosophical roots of Donna Haraway's cyborg imagery: descartes and Heidegger through Latour, Derrida, and Agamben. Hum. Stud. 37, 505–528. 10.1007/s10746-014-9327-z

[B37] Rizzolatti G. Fabbri-Destro M. (2008). The mirror system and its role in social cognition. Curr. Opin. in Neurobiol. 18, 179–184. 10.1016/j.conb.2008.08.001 18706501

[B38] Rockmore T. (1995). Heidegger and French Philosophy: Humanism, Anti-Humanism, and Being. London: Routledge.

[B39] Rothaar M. (2010). Human dignity and human rights in bioethics. The Kantian approach. Med. Health Care Philos. 13, 251–257. 10.1007/s11019-010-9249-0 20411338

[B40] Sabanovic S. (2014). Inventing Japan's robotics culture: the repeated assembly of science, technology, and culture in social robotics. Soc. Stud. Sci. 44, 342–367. 10.1177/0306312713509704 25051586

[B41] Sgreccia E. (2012). Personalist Bioethics: Foundations and Applications. Milan: Vita y Pensiero.

[B42] Waters B. (2006). From Human to Posthuman: Christian Theology and Technology in a Postmodern World. Aldershot: Ashgate, 31.

[B43] Wolpaw J. R. (2018). The negociated equilibrium model of spinal cord function. J. Physiol. 596, 3469–3491. 10.1113/JP275532 29663410 PMC6092289

[B44] Yuste R. (2010). Dendritic Spines. Cambridge, MA: MIT Press. 10.7551/mitpress/9780262013505.001.0001

